# Complementary therapy in palliative care: A synthesis of qualitative and quantitative systematic reviews

**DOI:** 10.1177/0269216320942450

**Published:** 2020-07-15

**Authors:** Megan Armstrong, Nuriye Kupeli, Kate Flemming, Patrick Stone, Susie Wilkinson, Bridget Candy

**Affiliations:** 1 Marie Curie Palliative Care Research Department, Division of Psychiatry, University College London, London, UK; 2 Department of Health Sciences, University of York, York, UK; 3 Palliative Care Institute, University of Liverpool, Liverpool, UK

**Keywords:** Complementary therapy, aromatherapy, massage, reflexology, palliative, systematic review, quality of life, synthesis

## Abstract

**Background:**

Interventions delivered in palliative care are complex and their evaluation through qualitative and quantitative research can lead to contrasting results. In a systematic review of trials, the effectiveness results of complementary therapies in palliative care were inconclusive; however, our qualitative synthesis showed participants perceived them to be beneficial.

**Aim:**

Use a novel methodology to synthesise evidence from qualitative and quantitative systematic reviews on complementary therapy in palliative care to explore the following: (1) If interventions delivered in trials reflect how participants in qualitative studies report they are delivered in real-life settings and (2) whether quality of life measures used in trials capture perceived benefits that are reported in qualitative studies.

**Methods:**

Two matrix tables were formulated. In one, key components in delivery of the complementary therapy from the qualitative synthesis which are as follows: (1) relationship with therapist, (2) comfortable environment, (3) choices (e.g. area of massage) and (4) frequent sessions, were plotted against intervention description, to explore matches and mismatches. In the other, items included in quality of life scales were compared with perceived benefits of complementary therapy.

**Results:**

None of the trials included all four key delivery components. The five quality of life scales used in the trials failed to capture the range of perceived benefits from the complementary therapies and many included inappropriate or redundant items.

**Conclusions:**

By integrating qualitative and quantitative review data, we determined the reasons trials may be inconclusive. This methodological exemplar provides a framework for understanding complexity in outcomes across trials and a direction for future research.

**What is already known about the topic?**

- A systematic review of effectiveness data on aromatherapy, massage and reflexology in palliative care drew inconclusive conclusions.
- A systematic review of qualitative evidences shows palliative care patients highly value complementary therapy.

**What this paper adds?**

- None of the aromatherapy, massage or reflexology trials included all key delivery components as outlined by palliative care patients.
- The five quality of life scales used in the trials failed to capture the range of perceived benefits from the complementary therapies and many included inappropriate or redundant items.
- This novel but simple method of integrating synthesised qualitative and quantitative reviews through matrices allows the reasons for inconclusive trial evidence to be explored.

**Implications for practice, theory or policy**

- This synthesis has highlighted a need for fully powered, robust trials of aromatherapy, massage and reflexology that are conducted with the key components described by people with palliative care needs. Outcome measures should be appropriate to capture the range of potential benefits highlighted by people with palliative needs. In the meantime, complementary therapies should continue to be offered as part of palliative care.

## Introduction

People with palliative care needs often seek complementary therapies in an attempt to reduce their physical symptom burden, help control treatment side effects and/or improve their psychological well-being.^1^ With tightening of financial constraints, there is an increasing need to justify provision through rigorous research on the impact of complementary therapies in people with palliative needs. We have previously published two systematic reviews on the following three complementary therapies commonly provided in palliative care: aromatherapy, massage and reflexology. Complementary therapy is commonly assessed by its benefits on quality of life; however, our systematic review on trial evidence found little effect on this.^2^ Nonetheless, our parallel review of qualitative evidence showed wide ranging perceived impact including improved well-being and hopefulness for the future.^3^ These two reviews clearly demonstrate a discrepancy between the benefits that people with palliative care needs report about complementary therapy and the lack of benefits found in clinical trials.

Interventions used in palliative care are often context dependent and complex in nature. They frequently involve multiple components designed to relieve suffering and improve well-being. These factors may explain why the findings of trials or synthesised evidence of trials are often inconclusive. The Medical Research Council’s guidelines on developing complex interventions highlight that a lack of effect may reflect implementation failure rather than ineffectiveness;^4^ therefore, reasons for a lack of effect should be explored.

Qualitative data can highlight which components of the interventions are, from the participants’ views, beneficial or unhelpful,^4^ as well as whether the participants found it enjoyable and why was that.^5^ The value of synthesising qualitative and quantitative findings in intervention development is widely recognised, as it allows researchers and clinicians to build interventions that are more acceptable to the target population.^6^ When there is noted inconsistency between the quantitative and the qualitative findings, there is a need to improve understanding and to inform future intervention development through an in-depth comparison of the data. Integrating qualitative and quantitative systematic reviews is seldom done; however, when it has been conducted, it has shown the value of using qualitative syntheses by enabling the development of an intervention that systematic reviews of trials could not do alone.^7^

One approach to integrating qualitative and quantitative findings is through matrices to explore overlap and gaps.^8^ Matrices can be used in evidence synthesis to explore components of interventions with reported benefits from qualitative data. Taking this further, it is also possible to explore individual items from the outcome measures used in the trials to determine whether they are appropriate for the target population and can accurately capture the benefits of the intervention.^9^ The limitations of quality of life scales in palliative care are well reported, with many scales not being thoroughly evaluated for validity^10^ and no scale appearing to cover all domains considered important by those in palliative care.^11^ Therefore, it is important to explore these outcome measures in detail within the context of complementary therapy in palliative care.

We report on a combined synthesis of data from a systematic review of 22 trials on complementary therapy, with a qualitative evidence synthesis of five studies exploring the views of patients who have received complementary therapy as part of palliative care treatment. To our knowledge, this integration of two systematic reviews on the qualitative and trial evidence is the first of its kind to explore both the components of interventions and the appropriateness of outcome measures. We believe that synthesising all available data, will lead to better informed recommendations about the role of complementary therapies in palliative care and in future research.

## Research synthesis questions

1. Do interventions being delivered in trials reflect how participants in qualitative studies report they are delivered in real-life settings?
2. Do the quality of life measures used in trials capture perceived benefits that are reported in qualitative studies?

## Methods

### Trial registration/protocol

The review protocol is registered on the PROSPERO database (22/11/2017 CRD42017081409). Available from: www.crd.york.ac.uk/PROSPERO/display_record.php?ID=CRD42017081409.

### Design and data sources

The two systematic reviews integrated in this article are ‘The effectiveness of aromatherapy, massage and reflexology in people with palliative care needs: A systematic review’^2^ and ‘Aromatherapy, massage and reflexology: A systematic review and thematic synthesis of the perspectives from people with palliative care needs’.^3^ From the systematic review on effectiveness, data were extracted on the delivery of the intervention and how the intervention was measured. From the systematic review of qualitative studies, synthesis data were extracted on the components of the intervention that the participants reported were important and the perceived benefits of the interventions. Data were then combined within matrix tables contrasting trial data with qualitative data.

### Systematic review on effectiveness

The systematic review approach followed Cochrane guidelines on the evaluation of evidence from trials on effectiveness^12^ and the Template for Intervention Description and Replication (TIDieR) was used to extract key features of the intervention.^13^ In total, 22 randomised controlled trials (RCTs) were included in the review. Eight trials evaluated aromatherapy,^14–21^ eight trials evaluated massage^22–29^ and six evaluated reflexology.^30–35^ The aims of these trials were to evaluate outcomes, such as quality of life, for people with palliative care needs using complementary therapy. Of the 22 trials, 20 were conducted in high-income countries. Most of the sample population had advanced cancer (15/22 trials) and were female (62%). The risk of bias in the trials was high, primarily due to a lack of blinding and small sample sizes. Due to heterogeneity of the trials, no meta-analysis was conducted and data were narratively synthesised instead.

### Systematic review of qualitative studies

Five relevant qualitative studies^36–40^ were synthesised using thematic synthesis methodology based on the guidelines by Thomas and Harden^41^ and were informed by guidance from the Cochrane Qualitative and Implementation Methods Group. Three studies focused on massage,^36,37,42^ one on aromatherapy^39^ and one on reflexology.^40^ The aims of these papers were to explore palliative patients’ perspectives of the benefits and harms of complementary therapy routinely delivered in palliative care settings. All studies were conducted in high-income countries and in the sample population, all had advanced cancer and were predominantly female (83%). Three analytical themes were identified: (1) Experience during the therapy (enhanced well-being and escapism), (2) beyond the complementary therapy session (lasting benefits and overall evaluation) and (3) delivery of complementary therapy in palliative care (value of the therapist and delivery of the complementary therapy). The quality of the qualitative papers was judged as reasonable to good.

Using the guidelines from the GRADE-CERQual (‘Confidence in the Evidence from Reviews of Qualitative research’),^43^ we assessed that we had no concerns about the methodology of the five primary papers or the coherence of the synthesised evidence from the primary studies. While there were only five primary studies, the data were rich enough to explore people with palliative needs’ opinions and experiences of complementary therapies. Finally, there were no concerns about the relevance of the primary evidence to the context of the review (i.e. palliative patients’ experiences of complementary therapy).

## Procedure

Our mixed synthesis method was informed by the Cochrane Qualitative and Implementation Methods Group’s guidance on integrating qualitative and quantitative review evidence.^44^ Specifically, to aid exploration of trial results, we selected the development of matrix tables as the appropriate tool. Tables were populated by listing findings from the qualitative synthesis and plotting data from the trials identified from the quantitative review as either a match or a mismatch. We developed two matrix tables to answer our research questions; the data extracted and how this was done are detailed below.

### Do interventions being delivered in trials reflect how participants in qualitative studies report they are delivered in real-life settings?

The following four key components of delivery of complementary therapy were identified from the qualitative synthesis as important: (1) building a relationship and interacting with the therapist, (2) being treated in a comfortable, familiar environment such as a hospice or at home, (3) having choices to enable a sense of control (e.g. choice of oils or area of massage) and (4) having an acceptable number of sessions. Complementary therapists suggest that a minimum of four sessions, delivered once a week (to allow sufficient recovery time), are required for therapies to have an effect;^20^ therefore, we have taken this as the minimum number of sessions to be acceptable. To create the matrix table, the four key intervention components were listed along the top and then information relating to intervention delivery was extracted from the 22 trial papers and plotted to reveal which trials included these components (indicated in green), which were unknown (yellow) and in which trials this component was not included (red).

### Do the quality of life measures used in trials capture perceived benefits identified in the qualitative synthesis?

To explore this research question, we developed a matrix by listing perceived benefits (or any harms) of complementary therapy identified from the qualitative synthesis and plotting each item from the quality of life scales to explore whether it was a match (indicated in green) or not (left blank). Quality of life was chosen as the most common outcome measure in the trials (*n* = 12/22 trials) and the only multi-domain scale on general well-being. There were five different multi-item scales used: McGill quality of life scale,^45^ Missoula-Vitas Quality Of Life Index,^46^ Quality of Life Inventory (QOLI),^47^ Functional Assessment of Cancer Therapy-Breast (FACT-B)^48^ and EuroQOL.^49^

The benefits extracted from the qualitative synthesis included the following: (1) improved well-being (relaxation, reducing physical symptoms, reducing negative thoughts, improved sleep, feeling empowered, feeling special/important and feeling dignified); (2) escapism and living in the present moment (feeling free from disease and worry, respite, altered/halted time, floating away/walking on air, living in the present moment and a feeling of inner peace); (3) long-term benefits (feeling good after the session, positive memories formed and providing hope for the future); (4) benefits from the therapist (developing a special relationship, receiving compassion, reduced loneliness, caring attention and opportunity to communicate with someone); and (5) overall benefits (positive and enjoyable experience and a reward for themselves). No harms were identified in the qualitative synthesis.

Making a judgement about whether items from the quality of life measures mapped on to perceived benefits was done independently by two authors (M.A. and B.C.) and any disagreements resolved through discussion.

## Results

### Do interventions that are delivered in trials reflect how participants in qualitative studies report they are delivered in real-life settings?

See Matrix Table 1.

**Table 1.** Comparing components of complementary therapy that palliative patients value with trial interventions.

Trial	Location of intervention	Number of intervention sessions	Providing a choice (e.g. oils or area of massage)	Therapist interaction
	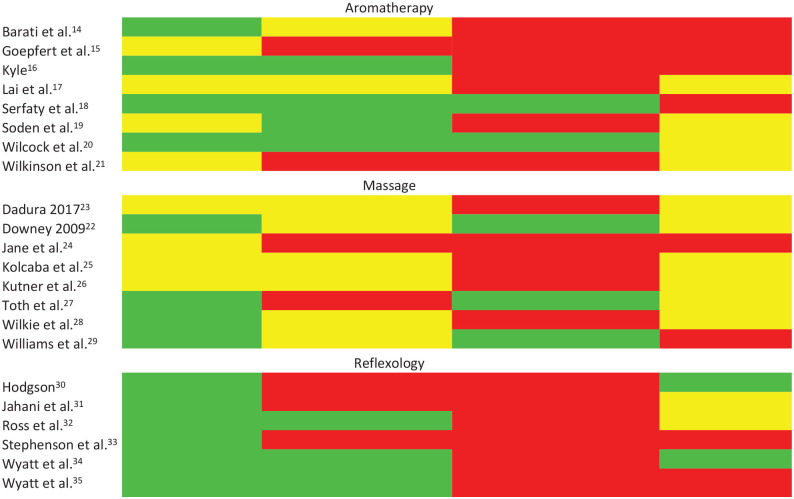			
Barati et al.^14^				
Goepfert et al.^15^				
Kyle^16^				
Lai et al.^17^				
Serfaty et al.^18^				
Soden et al.^19^				
Wilcock et al.^20^				
Wilkinson et al.^21^				

Dadura 2017^23^				
Downey 2009^22^				
Jane et al.^24^				
Kolcaba et al.^25^				
Kutner et al.^26^				
Toth et al.^27^				
Wilkie et al.^28^				
Williams et al.^29^				

Hodgson^30^				
Jahani et al.^31^				
Ross et al.^32^				
Stephenson et al.^33^				
Wyatt et al.^34^				
Wyatt et al.^35^				

Green indicates a match, yellow indicates uncertain and red indicates a mismatch.

## Aromatherapy

None of the eight trials’ intervention arms included all four key components. Four of the eight trials reported delivering therapy in a familiar or comfortable setting such as a hospice or at home.^14,16,18,20^ Two gave participants choice in the oils used in the massage^18,20^ and had at least four sessions of aromatherapy.^18–20^ None highlighted any focus on building a positive relationship with the therapist or encouraging interaction; indeed, in two trials there was no therapist involvement as therapy was self-performed^14,15^ and two other trials specified that such interaction was kept to a minimum.^18,19^

## Massage

None of the eight trials included all four components in their intervention. Four trials reported acceptable settings of therapy.^22,27–29^ No trial offered four or more sessions delivered weekly; six trials offered at least four sessions, but were often conducted in short succession (e.g. every day).^22–24,26,28,29^ Three provided some form of choice including massage location on the body, pressure and choice of oils.^22,27,29^ No trial reported encouraging interaction; two specified no interaction was allowed or encouraged.^24,29^

## Reflexology

None of the six trials included all four components in their intervention. All six reported acceptable settings of therapy. Three trials had four or more sessions offered weekly.^32,34,35^ None of the six trials gave participants choices over the intervention. Finally, two trials discussed promoting interaction between therapist and participants, while not giving any therapeutic advice^30,34^ and two trials were conducted by participants’ partners, which therefore gave no option for developing a relationship with someone outside of their immediate situation.^33,35^

### Do the quality of life measures used in trials capture perceived benefits that were found in the qualitative synthesis?

See Supplementary Matrix Table 2. None of the quality of life five scales meaningfully reflected the aspects of quality of life reported by participants in the qualitative synthesis. Out of 23 benefits identified in the qualitative review, 12 matched with items from 5 quality of life scales. However, only one item, a reduction in physical symptoms, came up in all five scales. Other benefits that mapped on to the scales were reducing negative thoughts (4/5), improved sleep (2/5), feeling empowered (1/5), feeling special (1/5), feeling dignified (1/5), being free from disease/worry (2/5), inner peace (1/5), feeling good (1/5), hope for the future (2/5), receiving compassion (1/5) and caring attention (1/5). The 11 benefits not captured by the scales were relaxation, respite, altering or halting time, floating away/walking on air, living in the present moment, inner peace, development of positive memories, developing a special relationship, reduced loneliness, opportunity to communicate, positive experiences and a feeling of personal reward.

There were 30 items from the five scales that were not found as a benefit of complementary therapies as described by people with palliative needs. These included satisfaction with sex, having affairs in order, achieving life goals, family accepting the illness, meeting family needs and being more satisfied with oneself as a person now than before illness. Overall, McGill Quality of Life Scale showed most overlap capturing 12 of 23 benefits with only two items not found as a benefit in qualitative synthesis.

## Discussion

Our synthesis of quantitative and qualitative systematic reviews has identified that complementary therapies, specifically aromatherapy, massage and reflexology, delivered in trial settings do not reflect the ways in which palliative care patients, who value these interventions, report they are provided. Furthermore, the quality of life measures frequently used to assess effectiveness of these therapies do not cover the domains that patients themselves indicate are most important. These findings suggest that it is premature to conclude that complementary therapies are ineffective in palliative care; rather, the evidence suggests that they have not yet been adequately evaluated.

People with palliative needs highlighted the value of the therapeutic relationship with the complementary therapist, the importance of having control over elements of their treatment, and the preference for regular sessions provided in a familiar and friendly environment. However, none of the 22 trials included all four of these components. The two components most commonly missing were participant–therapist interaction and availability of choice (e.g. choice of oils or location of massage); this is likely due to an understandable attempt to standardise interventions.

Standardising components, so each participant gets exactly the same intervention, is often seen as gold standard in trials.^50^ However, Medical Research Council guidelines on developing complex interventions highlight that strict standardisation may be inappropriate for certain interventions.^4^ Indeed, due to the emergence of more complex interventions, it is suggested that the function and process of the intervention should be standardised, but not the components themselves.^51^ In this context, complementary therapy interventions could be standardised, for instance, by offering participants a choice of oils and the option to interact with the therapist, so it is consistently tailored to their need. Removing key components of the intervention that have been identified as valuable by the intended beneficiaries runs the risk that studies will thereby fail to observe positive outcomes. Furthermore, conclusions based on trials that have evaluated therapies with artificial constraints, cannot necessarily be applied to bespoke complementary therapies delivered in real-life settings. At the same time, some clinical guidelines questioning the place of complementary therapies in palliative care because of lack of effectiveness.^52^

Another key finding from this synthesis was the inappropriateness of existing quality of life measures for capturing the range of benefits that people in palliative care reportedly experience when they engage in complementary therapy. Many of the benefits that participants reported in qualitative studies were not covered by the items in quality of life scales used in quantitative studies (e.g. improvement in sleep, hope for the future and reduction in loneliness). Moreover, some of the items from quality of life measures that were used were unlikely to be altered following complementary therapy; in particular, items that were unrelated to the therapy administered, such as financial affairs, having affairs in order and ability to spend time with family and friends. While these items may be important to someone’s quality of life, they are unlikely to be altered by complementary therapy; therefore, the scale becomes less sensitive to detect change.

## Strengths and limitations

This article is one of only a few studies in palliative care that have synthesised findings from qualitative and quantitative systematic reviews and, therefore, there is no ‘gold standard’ method. We used existing advocated approaches to ensure the transparency and replicability of our methods. Determining how quality of life scales and the benefits of complementary therapy were integrated within the matrices was somewhat subjective; however, we attempted to reduce the likelihood of this by having two reviewers complete the matrices independently.

In this article, we have reported a novel but straightforward way to integrate systematic reviews of effectiveness and qualitative evidence syntheses. By using this method, we have identified potential concerns with the intervention content and outcome measures of trials that otherwise would not have been identified. As policy decisions are often based on effectiveness evidence, this synthesis highlights the need to pool data from qualitative and quantitative research. We were able to synthesise the data with ease as we authored the two source reviews and, therefore, were fully submerged and familiar with the data. Although this is not to suggest this approach should not be considered by researchers who did not author the primary sources of the syntheses. The outcome of this endeavour is not definitive, rather a key aim; it is to provide a more informed direction for further intervention development.

## Future research and clinical implications

Future researchers should consider the benefits of combining quantitative and qualitative studies to provide more holistic conclusions regarding the purported benefits or effectiveness of treatments. Before attempting such a synthesis, it is important to ensure there are sufficient data and explore whether the qualitative studies are ‘trial sibling’ or ‘unrelated’ studies.^53^ Sibling studies have the same population as the trials allowing for confidence of integration of data; however, they do not provide ‘real-world’ data. Studies unrelated to the trials need to be checked to ensure they do not vary too much in terms of population characteristics. For instance, in this synthesis, all studies were unrelated, but the population from both reviews was predominantly female, with advanced cancer from high-income countries; therefore, the reviews were appropriate to integrate.

This synthesis has highlighted a need for fully powered, robust trials of aromatherapy, massage and reflexology that are conducted in accordance with the four key components of delivery described by people with palliative care needs. Furthermore, there is a need to use an appropriate outcome measure that can capture the range of potential benefits highlighted by people with palliative needs. Researchers should explore in more depth the aims of the complementary therapy (e.g. to provide a sense of relaxation rather than alter someone’s financial situation), which can be guided by the therapists and the patients, as well as how to capture benefits that happen during the session but may not last (e.g. a sense of floating away).

Due to the reported subjective benefits and lack of harms described in qualitative studies and the limitations of existing trials, we recommend that palliative care services continue to offer complementary therapy to patients as part of their holistic treatment, until more evidence is available.

## Conclusion

Trials exploring the effectiveness of aromatherapy, massage and reflexology have not delivered interventions in the way that they are routinely delivered in palliative care, making generalisation of findings to ‘real-world’ settings difficult. Furthermore, the quality of life outcome measures used in clinical trials are unable to capture the range of perceived benefits and may be insensitive to potential changes. Future research should develop and use more appropriate outcome measures and evaluate interventions that reflect real-world practice as closely as possible. Our novel approach has both highlighted and sought to address the impracticality of RCTs of complex interventions as the gold standard for ‘real-world’ situations, particularly as individualised as palliative care.

## Supplemental Material

Supplementary_table_1 – Supplemental material for Complementary therapy in palliative care: A synthesis of qualitative and quantitative systematic reviews Click here for additional data file. Supplemental material, Supplementary_table_1 for Complementary therapy in palliative care: A synthesis of qualitative and quantitative systematic reviews by Megan Armstrong, Nuriye Kupeli, Kate Flemming, Patrick Stone, Susie Wilkinson and Bridget Candy in Palliative Medicine

## References

[1] Verhoef M. Complementary and alternative approaches in palliative care: why are advanced cancer patients using them? Prog Palliat Care 2012; 20(5): 264–271.

[2] Candy B Armstrong M Flemming K, et al The effectiveness of aromatherapy, massage and reflexology in people with palliative care needs: a systematic review. Palliat Med 2020; 34: 179–194. 31659939 10.1177/0269216319884198 PMC7000853

[3] Armstrong M Flemming K Kupeli N, et al Aromatherapy, massage and reflexology: a systematic review and thematic synthesis of the perspectives from people with palliative care needs. Palliat Med 2019; 33(7): 757–769. 31060455 10.1177/0269216319846440 PMC6985994

[4] Craig P Dieppe P Macintyre S, et al Developing and evaluating complex interventions: the new Medical Research Council guidance. BMJ 2008; 337: a1655. 18824488 10.1136/bmj.a1655 PMC2769032

[5] Lobo MA Kagan SH Corrigan JD Research design options for intervention studies. Pediatr Phys Ther 2017; 29: S57–S63. 28654478 10.1097/PEP.0000000000000380 PMC5488684

[6] Flemming K The knowledge base for evidence-based nursing: a role for mixed methods research. ANS Adv Nurs Sci 2007; 30(1): 41–51. 17299283 10.1097/00012272-200701000-00005

[7] Flemming K Closs SJ Hughes ND, et al Using qualitative research to overcome the shortcomings of systematic reviews when designing of a self-management intervention for advanced cancer pain. Int J Qual Method 2016; 15(1): 670656.

[8] Candy B King M Jones L, et al Using qualitative evidence on patients’ views to help understand variation in effectiveness of complex interventions: a qualitative comparative analysis. Trials 2013; 14(1): 179. 23777465 10.1186/1745-6215-14-179 PMC3693880

[9] Moffatt S White M Mackintosh J, et al Using quantitative and qualitative data in health services research: what happens when mixed method findings conflict? BMC Heal Serv Res 2006; 6(1): 28. 10.1186/1472-6963-6-28 PMC1434735 16524479

[10] Albers G Echteld MA de Vet HC, et al Evaluation of quality-of-life measures for use in palliative care: a systematic review. Palliat Med 2010; 24(1): 17–37. 19843620 10.1177/0269216309346593

[11] McCaffrey N Bradley S Ratcliffe J, et al What aspects of quality of life are important from palliative care patients’ perspectives? A systematic review of qualitative research. J Pain Symptom Manage 2016; 52(2): 318–328. 27216362 10.1016/j.jpainsymman.2016.02.012

[12] Higgins J Green S Cochrane handbook for systematic reviews of interventions. The Cochrane Collaboration, 2011, https://training.cochrane.org/handbook/archive/v5.1/

[13] Hoffmann TC Galsziou PR Milne R, et al Better reporting of interventions: template for intervention description and replication (TIDieR) checklist and guide. BMJ 2014; 348: g1687. 24609605 10.1136/bmj.g1687

[14] Barati F Nasiri A Akbari N, et al The effect of aromatherapy on anxiety in patients. Nephrourology 2016; 8(5): e38347. 10.5812/numonthly.38347 PMC5111093 27878109

[15] Goepfert M Liebl P Herth N, et al Aroma oil therapy in palliative care: a pilot study with physiological parameters in conscious as well as unconscious patients. J Cancer Res Clin Oncol 2017; 143(10): 2123–2129. 28634728 10.1007/s00432-017-2460-0 PMC11819024

[16] Kyle G Evaluating the effectiveness of aromatherapy in reducing levels of anxiety in palliative care patients: results of a pilot study. Complement Ther Clin Pract 2006; 12(2): 148–155. 16648093 10.1016/j.ctcp.2005.11.003

[17] Lai TKT Cheung MC Lo CK, et al Effectiveness of aroma massage on advanced cancer patients with constipation: a pilot study. Complement Ther Clin Pract 2011; 17(1): 37–43. 21168113 10.1016/j.ctcp.2010.02.004

[18] Serfaty M Wilkinson S Freeman C, et al The ToT study: helping with Touch or Talk (ToT): a pilot randomised controlled trial to examine the clinical effectiveness of aromatherapy massage versus cognitive behaviour therapy for emotional distress in patients in cancer/palliative care. Psycho-Oncol 2012; 21(5): 563–569. 10.1002/pon.1921 21370309

[19] Soden K Vincent K Craske S, et al A randomized controlled trial of aromatherapy massage in a hospice setting. Palliat Med 2004; 18(2): 87–92. 15046404 10.1191/0269216304pm874oa

[20] Wilcock A Manderson C Weller R, et al Does aromatherapy massage benefit patients with cancer attending a specialist palliative care day centre. Palliat Med 2004; 18(4): 287–290. 15198118 10.1191/0269216304pm895oa

[21] Wilkinson S Aldridge J Salmon I, et al An evaluation of aromatherapy massage in palliative care. Palliat Med 1999; 13(5): 409–417. 10659113 10.1191/026921699678148345

[22] Downey L Diehr P Standish LJ, et al Might massage or guided meditation provide ‘means to a better end’? Primary outcomes from an efficacy trail with patients at the end of life. J Palliat Care 2009; 25(2): 100–108. 19678461 PMC2858762

[23] Dadura E Stępień P Iwańska D, et al Effects of abdominal massage on constipation in palliative care patients: a pilot study. Adv Rehabil 2017; 31(4): 19–34.

[24] Jane S-W Chen SL Wilkie DJ, et al Effects of massage on pain, mood status, relaxation, and sleep in Taiwanese patients with metastatic bone pain: a randomized clinical trial. Pain 2011; 152(10): 2432–2442. 21802850 10.1016/j.pain.2011.06.021

[25] Kolcaba K Dowd T Steiner R, et al Efficacy of hand massage for enhancing the comfort of hospice patients. J Hosp Palliat Nurs 2004; 6: 91–102.

[26] Kutner J Smith MC Corbing L, et al Massage therapy versus simple touch to improve pain and mood in patients with advanced cancer: a randomized trial. Ann Inter Med 2008; 149: 369–379. 10.7326/0003-4819-149-6-200809160-00003 PMC2631433 18794556

[27] Toth M Marcantonio ER Davis RB, et al Massage therapy for patients with metastatic cancer: a pilot randomized controlled trial. J Altern Complement Med 2013; 19(7): 650–656. 23368724 10.1089/acm.2012.0466 PMC3700435

[28] Wilkie DJ Kampbell J Cutshall S, et al Effects of massage on pain intensity, analgesics and quality of life in patients with cancer pain: a pilot study of a randomized clinical trial conducted within hospice care delivery. Hosp J 2000; 15(3): 31–53. 11315685

[29] Williams A-L Selwyn PA Liberti L, et al A randomized controlled trial of meditation and massage effects on quality of life in people with late-stage disease: a pilot study. J Palliat Med 2005; 8(5): 939–952. 16238507 10.1089/jpm.2005.8.939

[30] Hodgson H Does reflexology impact on cancer patients’ quality of life? Nurs Stand 2000; 14: 33–38. 10.7748/ns2000.04.14.31.33.c2817 11973949

[31] Jahani S Salari F Elahi N, et al Investigating the effect of reflexology in intensity of pain and anxiety among patients suffering from metastatic cancer in adults’ hematology ward. Asian Journal of Pharmaceutical and Clinical Research 2018; 11(6): 401–405.

[32] Ross C Hamilton J Macrae G, et al A pilot study to evaluate the effect of reflexology on mood and symptom rating of advanced cancer patients. Palliat Med 2002; 16(6): 544–545. 12465705 10.1191/0269216302pm597xx

[33] Stephenson NL Swanson M Dalton J, et al Partner-delivered reflexology: effects on cancer pain and anxiety. Oncol Nurs Forum 2007; 34(1): 127–132. 17562639 10.1188/07.ONF.127-132

[34] Wyatt G Sikorskii A Rahbar MH, et al Health-related quality-of-life outcomes: a reflexology trial with patients with advanced-stage breast cancer. Oncol Nurs Forum 2012; 39(6): 568–577. 23107851 10.1188/12.ONF.568-577 PMC3576031

[35] Wyatt G Sikorskii A Tesnjak I, et al A randomized clinical trial of caregiver-delivered reflexology for symptom management during breast cancer treatment. J Pain Sympt Manage 2017; 54: 670–679. 10.1016/j.jpainsymman.2017.07.037 PMC5650941 28743659

[36] Beck I Runeson I Blomqvist K To find inner peace: soft massage as an established and integrated part of palliative care. Int J Palliat Nurs 2009; 15(11): 541–545. 20081728 10.12968/ijpn.2009.15.11.45493

[37] Billhult A Dahlberg K A meaningful relief from suffering experiences of massage in cancer care. Cancer Nurs 2001; 24(3): 180–184. 11409061

[38] Cronfalk BS Strang P Ternestedt BM, et al The existential experiences of receiving soft tissue massage in palliative home care: an intervention. Support Care Cancer 2009; 17(9): 1203–1211. 19184127 10.1007/s00520-008-0575-1

[39] Dunwoody L Smyth A Davidson R Cancer patients’ experiences and evaluations of aromatherapy massage in palliative care. Int J Palliat Nurs 2002; 8(10): 497–504. 12419989 10.12968/ijpn.2002.8.10.10696

[40] Gambles M Crooke M Wilkinson S Evaluation of a hospice based reflexology service: a qualitative audit of patient perceptions. Eur J Oncol Nurs 2002; 6(1): 37–44. 12849608 10.1054/ejon.2001.0157

[41] Thomas J Harden A Methods for the thematic synthesis of qualitative research in systematic reviews. BMC Med Res Methodol 2008; 8(1): 45. 18616818 10.1186/1471-2288-8-45 PMC2478656

[42] Cronfalk BS Strang P Ternestedt BM Inner power, physical strength and existential well-being in daily life: relatives’ experiences of receiving soft tissue massage in palliative home care. J Clin Nurs 2009; 18(15): 2225–2233. 19583654 10.1111/j.1365-2702.2008.02517.x

[43] Lewin S Booth A Glenton C, et al Applying GRADE-CERQual to qualitative evidence synthesis findings: introduction to the series. Implement Sci 2018; 13: 10. 29384079 10.1186/s13012-017-0688-3 PMC5791040

[44] Harden A Thomas J Cargo M, et al Cochrane Qualitative and Implementation Methods Group guidance series – paper 5: methods for integrating qualitative and implementation evidence within intervention effectiveness reviews. J Clin Epidemiol 2018; 97: 70–78. 29242095 10.1016/j.jclinepi.2017.11.029

[45] Cohen SR Mount BM Strobel MG, et al The McGill Quality of Life Questionnaire: a measure of quality of life appropriate for people with advanced disease. Palliat Med 1995; 9(3): 207–219. 7582177 10.1177/026921639500900306

[46] Schwartz CE Merriman MP Reed G, et al Evaluation of the Missoula-VITAS Quality of Life Index – revised: research tool or clinical tool? J Palliat Med 2005; 8(1): 121–135. 15662181 10.1089/jpm.2005.8.121

[47] Frisch MB Cornell J Villanueva M, et al Clinical validation of the Quality of Life Inventory: a measure of life satisfaction for use in treatment planning and outcome assessment. Psychol Assess 1992; 4(1): 92–102.

[48] Brady MJ Cella DF Mo F, et al Reliability and validity of the functional assessment of cancer therapy-breast quality-of-life instrument. J Clin Oncol 1997; 15(3): 974–986. 9060536 10.1200/JCO.1997.15.3.974

[49] EuroQol Group. EuroQol-a new facility for the measurement of health-related quality of life. Heal Pol 1990; 16(3): 199–208. 10.1016/0168-8510(90)90421-9 10109801

[50] McMahon AD Study control, violators, inclusion criteria and defining explanatory and pragmatic trials. Stat Med 2002; 21(10): 1365–1376. 12185890 10.1002/sim.1120

[51] Hawe P Shiell A Riley T Complex interventions: how ‘out of control’ can a randomised controlled trial be? BMJ 2004; 328(7455): 1561–1563. 15217878 10.1136/bmj.328.7455.1561 PMC437159

[52] Preen C NICE to remove CAM from palliative care guidelines, http://www.complementaryhealthprofessionals.co.uk/#!NICE-to-remove-CAM-from-Palliative-Care-Guidelines/c1tla/56aa2b480cf289b6a281e14c2006

[53] Noyes J Hendry M Lewin S, et al Qualitative ‘trial-sibling’ studies and ‘unrelated’ qualitative studies contributed to complex intervention reviews. J Clin Epidemiol 2016; 74: 133–143. 26775628 10.1016/j.jclinepi.2016.01.009

